# Temporal assessment of neonatal pain after airway aspiration

**DOI:** 10.5935/0103-507X.20200011

**Published:** 2020

**Authors:** Isabelle Leandro Gimenez, Rafaella Fintelman Rodrigues, Marcella Campos de Faria Oliveira, Beatriz Alves Rezende Santos, Vanessa da Silva Neves Moreira Arakaki, Rosana Silva dos Santos, Rodrigo Tosta Peres, Clemax Couto Sant’Anna, Halina Cidrini Ferreira

**Affiliations:** 1 Universidade Federal do Rio de Janeiro - Rio de Janeiro (RJ), Brazil.; 2 Department of Physical Therapy, Faculdade de Medicina, Universidade Federal do Rio de Janeiro - Rio de Janeiro (RJ), Brazil.; 3 Department of Mathematics, Centro Federal de Educação Tecnológica Celso Suckow da Fonseca - Rio de Janeiro (RJ), Brazil.; 4 Department of Pediatrics, Faculdade de Medicina, Universidade Federal do Rio de Janeiro - Rio de Janeiro (RJ), Brazil.

**Keywords:** Pain, Pain measurement, Suction, Infant, premature, Surveys and questionnaires, Reproducibility of results, Dor, Medição da dor, Sucção, Recém-nascido prematuro, Inquéritos e questionários, Reprodutibilidade dos testes

## Abstract

**Objective:**

To temporally assess a painful stimulus in premature infants using 3 neonatal pain scales.

**Methods:**

A total of 83 premature infants were observed during airway aspiration by 3 evaluators (E1, E2 and E3) using 3 pain assessment scales (Neonatal Facial Coding System - NFCS; Neonatal Infant Pain Scale - NIPS; and Premature Infant Pain Profile - PIPP) at 5 time points: T1 (before airway aspiration), T2 (during airway aspiration), T3 (1 minute after airway aspiration), T4 (3 minutes after airway aspiration), and T5 (5 minutes after airway aspiration). Light’s Kappa (agreement among examiners and among scales at each time point) and the McNemar test (comparison among time points) were used considering p < 0.05.

**Results:**

There was a significant difference between the 3 examiners for T1 and T2 using the 3 scales. In T3, pain was observed in 22.9%/E1, 28.9%/E2, and 24.1%/E3 according to the NFCS; 22.9%/E1, 21.7%/E2, and 16.9%/E3 according to the NIPS; and 49.4%/E1, 53.9%/E2, and 47%/E3 according to the PIPP. There was a difference between T1 and T3 using the 3 scales, except for 2 examiners for the PIPP (E2: p = 0.15/E3: p = 0.17). Comparing T4 and T5 to T1, there was no difference in the 3 scales.

**Conclusion:**

Premature infants required at least 3 minutes to return to their initial state of rest (no pain).

## Abstract (pt)

**Objetivo:**

Avaliar temporalmente o estímulo doloroso em prematuros com o uso de três escalas de mensuração de dor neonatal.

**Métodos:**

Foram observados 83 prematuros durante a aspiração de vias aéreas por três avaliadores (E1, E2 e E3) utilizando três escalas de avaliação da dor (Neonatal Facial Coding System - NFCS; Neonatal Infant Pain Scale - NIPS; e Premature Infant Pain Profile - PIPP) em cinco momentos: T1 (antes da aspiração de vias aéreas), T2 (durante a aspiração de vias aéreas), T3 (1 minuto após a aspiração de vias aéreas), T4 (3 minutos após a aspiração de vias aéreas) e T5 (5 minutos após a aspiração de vias aéreas). Utilizaram-se o Light’s Kappa (concordância entre examinadores e entre as escalas em cada tempo) e teste de McNemar (comparação entre os tempos), considerando-se p < 0,05.

**Resultados:**

Houve diferença significativa entre T1 e T2 para os três examinadores nas três escalas. Em T3, observou-se dor em 22,9%/E1, 28,9%/E2 e 24,1%/E3 de acordo com a NFCS; 22,9%/E1, 21,7%/E2 e 16,9%/E3, conforme a NIPS e 49,4%/E1, 53,9%/E2 e 47%/E3 considerando a PIPP dos prematuros. Houve diferença entre T1 e T3 nas três escalas, exceto para dois examinadores na PIPP (E2: p = 0,15/ E3: p = 0,17). Ao comparar T4 e T5 ao T1, não houve diferença em nenhuma das três escalas.

**Conclusão:**

Os prematuros necessitaram de pelo menos 3 minutos para retornarem ao seu estado inicial de repouso (sem dor).

## INTRODUCTION

Pain is defined as an unpleasant sensory and emotional experience associated with actual tissue damage, potential or described, that is always subjective.^(1)^ However, this concept cannot be applied literally to newborns because of their lack of verbal ability and the absence of previous painful experiences, which would enable the comparison and description of the pain sensation.^(2)^

Pain is inherent to care in the neonatal intensive care unit (ICU) because numerous procedures and routine interventions are performed, with an average of 51 painful stimuli in only 1 day, including punctures and aspirations.^(3)^ Thus, professionals working in the neonatal ICU are increasingly concerned with measuring pain sensations related to manipulation because it is understood that the central nervous system (CNS) of newborns, including premature infants, is mature regarding painful stimuli.^(2)^

Prolonged exposure to pain may result in changes in the conformation of the brain, with consequent impaired development.^(4)^ The more premature a newborn is, the stronger the responses and the greater sensitivity to pain.^(5)^ The approach related to pain reduction in the early stages of childhood should be intensified to avoid future impairments, such as emotional, behavioral, learning and growth changes.^(6,7)^

One of the most important questions in this field of knowledge concerns the difficulty of assessing and measuring pain in newborns, the greatest obstacles for the appropriate treatment of pain in neonatal ICUs. There are many scales, but none of them emerges as the gold standard for evaluations.^(8)^

In addition, some non-pharmacological strategies have been proposed to reduce pain during various ICU procedures, including positioning in the bed and stimulus to non-nutritive sucking, among others.^(9,10)^ However, these are routinely performed by each institution and do not focus on the time of use. The study of the time neonates remain in pain after a given stimulus has not been performed, possibly resulting in actions that are ineffective or cause excessive manipulation.^(11,12)^

In this context, in addition to recognizing pain, it is essential to know for how long premature infants remain with painful sensations. With this, care teams can develop strategies based on concrete data. A temporal study with more than 1 scale and more than 1 evaluator is necessary so that, in the absence of a gold standard, the data are more reliable and are able to provide information about the possibility of 1 of the scales bringing better agreement among examiners.

The objective of the present study was to temporally evaluate a painful stimulus in premature infants using 3 neonatal pain scales.

## METHODS

Clinically stable premature infants with no diagnosis of neurological abnormality were included. The following newborn patients were excluded from the study: newborns with genetic syndromes and congenital diseases; newborns with altered transfontanelle ultrasonography after birth, using sedation or neuromuscular blockers and whose mothers had used illicit drugs or alcohol during pregnancy; newborns with Apgar score < 7 in the first minute and who did not recover after 5 minutes of life; newborns with conditions that cause pain, such as necrotizing enterocolitis and the presence of a thoracic or abdominal drain; and newborns using glycosylated solutions for at least 30 minutes before the beginning of the observations.

Three evaluators (E1, E2 and E3) observed premature infants initially at 3 time points: before (T1), during (T2), and 1 minute (T3) after airway aspiration. Considering a partial analysis of the results (n = 50), the team observed that 1 minute after the airway aspiration procedure was not enough time for the newborn to return to the initial state without pain. From this, the observation and completion of pain scales were performed at 5 time points (n = 33; total n = 83), namely, before the procedure (T1), during the procedure (T2), and 1 minute (T3), 3 minutes (T4), and 5 minutes (T5) after airway cleansing, using 3 pain assessment scales (Neonatal Facial Coding System - NFCS, Neonatal Infant Pain Scale - NIPS, and Premature Infant Pain Profile - PIPP) simultaneously. Airway aspiration was used as a control procedure because this technique generates severe pain.^(12,13)^ The professional who performed airway cleansing was always the same to avoid technical differences during manipulation, and observations were made during routine care.

Descriptive statistics were obtained. The analysis of agreement among the 3 evaluators at the time points cited for each pain scale was performed using Light’s Kappa test. The indicators of neonatal pain among the time points analyzed were compared using the McNemar test considering p ≤ 0.05 as statistically significant.

This was an observational study with a quantitative approach performed between March 2015 and May 2017, approved by the Ethics and Research Committee of a national public reference hospital (*Maternidade Escola* of the *Universidade Federal do Rio de Janeiro*), under number CAAE: 25211913.9.0000.5275.

## RESULTS

A total of 83 newborns with gestational age between 140 and 260 days (218.3 ± 24 days; Apgar score at the fifth minute ≥ 7) without sedation were included in the study. The Apgar score varied in the first minute from 4 to 9, with a median value of 7, and in the fifth minute from 7 to 10, with a median of 9. Regarding the ventilatory support in use at the time of data collection, 48 newborns (58%) were under continuous positive airway pressure (CPAP), and 6 (7%) were using an orotracheal tube. The others were not using any support, totaling 29 (35%) newborns breathing room air.

The 3 evaluators each filled out a form with all the necessary items for the 3 pain scales: NFCS, NIPS and PIPP. The total score for each scale was subsequently derived only by the principal investigator. Scores corresponding to the presence of pain were made according to each of the scales used. The descriptive analysis of these findings shows the percentage of newborns with or without pain in the first 3 time points analyzed for the 83 neonates (Table 1).

**Table 1:** Newborns with pain and no pain at T1, T2 and T3 for each scale and evaluator

	NFCS			NIPS			PIPP		
	E1	E2	E3	E1	E2	E3	E1	E2	E3
T1 without pain	94	91.6	97.6	95.2	95.2	98.8	63.9	54.2	61.4
T1 with pain	6	8.4	2.4	4.8	4.8	1.2	36.1	45.8	38.6
T2 without pain	1.2	2.4	2.4	9.6	6	7.2	0	3.6	1.2
T2 with pain	98.8	97.6	97.6	90.4	94	92.8	100	96.4	98.8
T3 without pain	77.1	71.1	75.9	77.1	78.3	83.1	50.6	47	53
T3 with pain	22.9	28.9	24.1	22.9	21.7	16.9	49.4	53	47

NFCS - Neonatal Facial Coding System; NIPS - Neonatal Infant Pain Scale; PIPP - Premature Infant Pain Profile; E1 - examiner 1; E2 - examiner 2; E3: examiner 3; T1 - analysis performed before airway aspiration; T2 - analysis performed during airway aspiration; T3 - analysis performed 1 minute after airway aspiration. The results are expressed as %.

**Figure 1:**
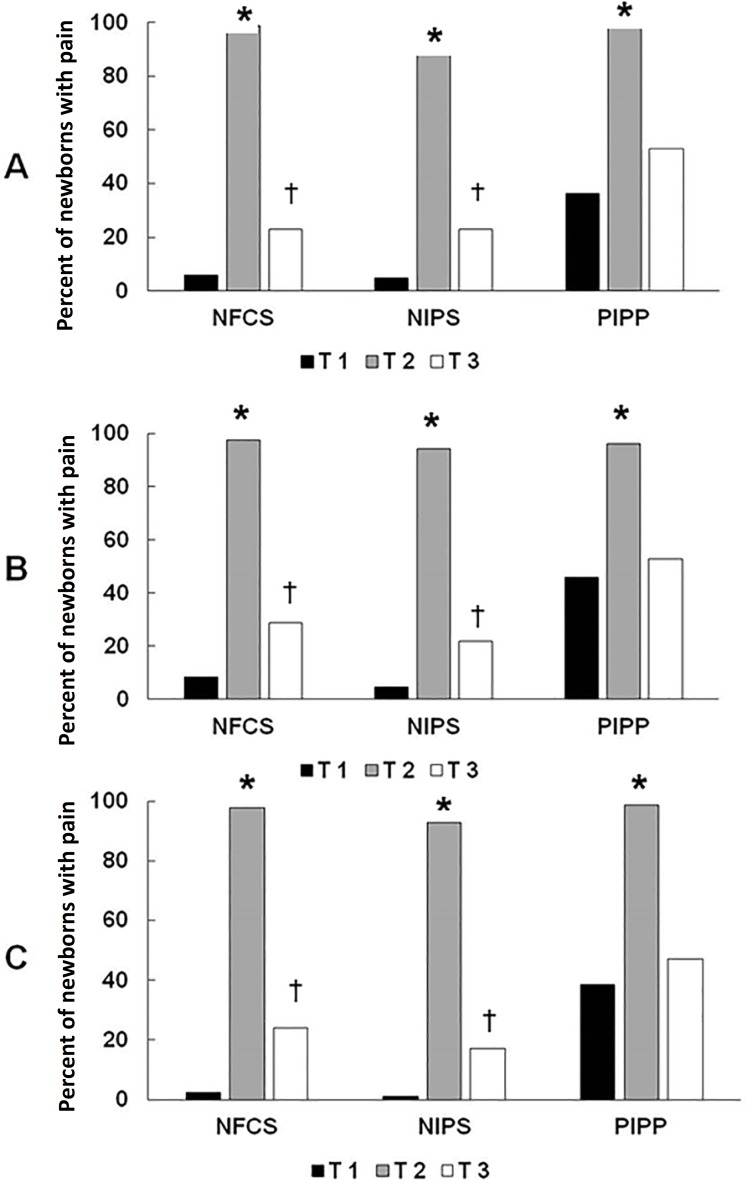
Temporal response to painful stimuli in premature infants per evaluator in the first 3 observation time points (n = 83). (A) Pain percentage assessed by the first evaluator using 3 scales; (B) Pain percentage assessed by the second evaluator using 3 scales; (C) Pain percentage assessed by the third evaluator using 3 scales. NFCS - Neonatal Facial Coding System; NIPS - Neonatal Infant Pain Scale; PIPP - Premature Infant Pain Profile; T1 - analysis performed before airway aspiration; T2 - analysis performed during airway aspiration; T3 - analysis performed 1 minute after airway aspiration (n = 83). *Statistically significant difference between T2 and time points T1 and T3; †statistically significant difference between T1 and T3. There was no difference between time points T1 and T3 on the PIPP scale (the McNemar test, with p <0.05 as statistically significant).

Based on the results in figure 1, the stimulus performed at T2 was potentially painful according to the 3 pain scales, and at T3 (1 minute after the procedure), there was still a percentage of pain greater than that at T1, which suggests that the time needed for newborn recovery was greater. There was a statistically significant difference between the T1 and T2 values for the 3 scales, as well as between T2 and T3. The p-value between time points T1 and T3 was analyzed to confirm that 1 minute was sufficient for newborn recovery; the results showed no difference between them only for 2 evaluators using the PIPP scale. Thus, in most cases, the newborn did not return to the initial stage.

From these first results, it was decided to increase the observation time to 2 more time points, T4 (3 minutes after the stimulus) and T5 (5 minutes after the stimulus) (n = 30). There was no significant difference when comparing these time points to T1; that is, they can be considered equal (Table 2). Thus, 3 minutes after the painful procedure was sufficient time for newborns to return to their initial state (T1).

**Table 2:** P-values for T1/T4 and T1/T5 for the 3 scales (n = 30)

	NFCS			NIPS			PIPP		
	E1	E2	E3	E1	E2	E3	E1	E2	E3
T1/T4									
p value	0.34	0.72	0.37	0.13	0.18	0.13	0.61	1	0.8
T1/T5									
p value	1	0.72	1	1	1	0.48	0.3	0.65	1

NFCS - Neonatal Facial Coding System; NIPS - Neonatal Infant Pain Scale; PIPP - Premature Infant Pain Profile; E1 - examiner 1; E2 - examiner 2; E3: examiner 3. p values ≤ 0.05 were considered statistically significant by the McNemar test.

Scales exhibited low agreement at all time points (Table 3). Time points T4 and T5 were not part of the agreement test because of the small sample size (n = 30). The same test was performed to observe the agreement among the evaluators (Table 3) in the first 3 data collection time points, and as a result, there was weak agreement among them.

**Table 3:** Values of agreement among the pain scales in the first 3 assessment time points (A) and among examiners (B)

	T1	T2	T3
A			
NFCS	0.05	-0.02	0.64
NIPS	0.33	0.27	0.68
PIPP	0.55	-0.01	0.62
B			
E1	0.32	0.07	0.54
E2	0.21	0.24	0.5
E3	0.26	0.2	0.45

NFCS - Neonatal Facial Coding System; NIPS - Neonatal Infant Pain Scale; PIPP - Premature Infant Pain Profile; T1 - analysis performed before airway aspiration; T2 - analysis performed during airway aspiration; T3 - analysis performed 1 minute after airway aspiration; E - examiner (n = 83). The analysis of agreement among the 3 evaluators at the time points indicated for each pain scale was performed using Light's Kappa test.

## DISCUSSION

The present study confirms airway aspiration as a potentially painful procedure.^(12,13)^ The time to return to the initial state (no pain) occurred after 3 minutes. From the weak agreement among the evaluators and scales, it was possible to corroborate the difficulty in proposing a gold standard for the evaluation of neonatal pain.

In the literature, there is a lack of studies that focus on the recovery time of newborns after painful stimuli. Aguilar Cordero et al.^(14)^ compared healthy newborns and newborns with Down syndrome in relation to the time needed to perceive a painful stimulus and then to recover. As a result, the study showed that children with Down syndrome require more time to recover from a painful stimulus. In turn, Campbell-Yeo et al.^(15)^ compared premature twins in the same incubator and in different incubators after heel puncture and concluded that those in the same incubator had a shorter recovery time after the painful stimulus than did twins in separate incubators. The present study is innovative in that it provides data on preterm infants evaluated temporally after a painful stimulus. Initially, only 3 time points were chosen for observation and analysis (T1, T2 and T3), as the researchers considered the possibility that 1 minute was sufficient for the return of newborns to their initial state. With data collection and partial data summarization, 1 minute was not enough time for newborns to stop feeling pain. Thus, from that moment, we extended the observation time to the third (T4) and fifth (T5) minutes after airway aspiration. With this, the results showed that at least 3 minutes is necessary for newborns to return to their initial state, without pain (T1).

The verification of the time needed for infants to recover after a painful stimulus is relevant and urgent because, from this perspective, it is possible to more objectively manage nonpharmacological and/or pharmacological strategies for neonatal pain relief after diverse procedures used in the neonatal ICU, which are known to cause discomfort. Protocols can be constructed in a safe and informed way, thus avoiding future sequelae that may occur in infants exposed to pain during neonatal hospitalization, such as emotional, behavioral, learning and growth impairment.^(6,7,16)^ Within this context, the present study confirms the findings of the literature, which cite airway aspiration as a stimulus that causes pain.^(12,13)^ Maximum scores were found for the 3 scales completed by the 3 evaluators for the occurrence of pain, which suggests that this technique must be carefully indicated and that routine use should not be conducted without responsible and technical assessment.

With regard to pain scales, in the present study, we chose to work with 3 scales that quantify pain in different ways, as there is no gold standard for these measurements. The NFCS is a scale that evaluates only facial expressions, i.e., it is a 1-dimensional scale. This scale excludes any physiological factor from its score.^(4,6,17)^ In turn, NIPS is a multidimensional scale that includes, in addition to facial expressions, 3 physiological items (crying, breathing pattern and state of arousal).^(4,6,17)^ The PIPP is a more comprehensive multidimensional scale because, in addition to physiological and behavioral parameters, it considers gestational age at birth.^(18,19)^ According to the American Academy of Pediatrics (AAP),^(20)^ only 5 scales underwent rigorous psychometric tests, including the NFCS and PIPP, which justifies choosing these scales. Regional criteria were used in the choice of the NIPS scale, as a previous publication related to this study (mapping of the knowledge of physical therapists in Rio de Janeiro -http://objdig.ufrj.br/50/teses/m/CCS_M_871761.pdf) showed that it was the most widely used scale in municipality care.

The PIPP scale is widely recommended in several studies in the literature;^(21)^ however, during its use in this study, the scale was, in fact, very comprehensive, but it was not easy to apply to clinical practice.^(12,21)^ This is because its score is based on a percentage of time and is more time consuming to calculate, which may be difficult to use daily at the bedside. However, for academic purposes, the PIPP scale is functional and complete. With the NFCS and NIPS, greater functionality was observed with regard to frequent use in the neonatal ICU.^(4,6,21)^ Their simple scoring methods make the measurement more agile but not less effective. Moreover, both showed very similar percentages of pain or no pain, which leads us to assume that the low agreement found among the 3 scales may have occurred because of the PIPP, which showed more discrepant values. Another possible explanation for the low agreement is that although the percentage of painful responses was similar between the scales and the evaluators (Table 1; Figure 1), the agreement was low (Tables 2 and 3). This can be explained by the characteristics of Light’s Kappa test, which, in the presence of many equal values for the same variable, considers the hypothesis that there may be false positives or false negatives, which reduces the final value.

By continuing to observe the low agreement among the scales and among the evaluators, the difficulty of proposing a gold standard scale for this type of analysis could be understood with greater clarity. The cause of this low interrater agreement may also be related to the low familiarity of the professionals with these instruments and, therefore, to not paying attention to pain perception in newborns. This topic should therefore be extended to all professionals who work directly with newborn manipulation at the bedside so they can understand neonatal care reality in relation to pain and be systematically trained to pay attention to the patient and the application of the scales. In the literature, there is a shortage of studies that address this topic, the majority of which are related to nursing, do not present systematization of pain management, and suggest the need for the implementation of classes and courses for care teams.^(22-25)^

## CONCLUSION

It took at least 3 minutes for newborns to recover from a painful stimulus and return to their initial state. Moreover, agreement between the scales and the examiners was weak, which confirms the absence of a gold standard for the assessment of pain in neonates and the challenge of systematizing this type of analysis.

In addition, including a minimum of 3 minutes in non-pharmacological strategies to combat neonatal pain is further explored, in addition to the inclusion of biological markers linked to stress, which can confirm and establish correlations with the visual scales, in the search for a gold standard measure.
